# Uso Atual de Ressonância Magnética Cardíaca Pediátrica no Brasil

**DOI:** 10.36660/abc.20190860

**Published:** 2021-02-19

**Authors:** Marcelo Felipe Kozak, Jorge Yussef Afiune, Lars Grosse-Wortmann

**Affiliations:** 1 Instituto de Cardiologia do Distrito FederalBrasíliaDFBrasilInstituto de Cardiologia do Distrito Federal - Cardiologia Pediátrica, Brasília, DF - Brasil; 2 Hospital da Criança de Brasília José de AlencarBrasíliaDFBrasilHospital da Criança de Brasília José de Alencar – Ecocardiografia, Brasília, DF - Brasil; 3 Doernbecher Children’s HospitalOregon Health and Science UniversityDepartment of PediatricsPortlandOregonEUADoernbecher Children’s Hospital, Oregon Health and Science University - Division of Pediatric Cardiology, Department of Pediatrics, Portland, Oregon - EUA; 4 Hospital for Sick ChildrenUniversity of TorontoDepartment of PediatricsTorontoOntarioCanadáThe Hospital for Sick Children, University of Toronto - Department of Pediatrics, Toronto, Ontario – Canadá

**Keywords:** Pediatria, Cardiopatias Congênitas/cirurgia, Técnicas e Procedimentos Diagnósticos, Imagem por Ressonância Magnética, Cateterismo Cardíaco

## Abstract

**Fundamento:**

Dados sobre o uso de ressonância magnética cardíaca (RMC) em crianças no Brasil são escassos.

**Objetivos:**

Buscamos oferecer informações sobre as práticas atuais de RMC pediátricas no Brasil.

**Métodos:**

Um questionário foi enviado a médicos solicitantes de RMC de todo o país, cobrindo informações sobre si próprios, sobre seus serviços de RMC, contexto clínico dos pacientes e sobre os obstáculos para a realização de RMC em crianças. Para a análise estatística, um p < 0,05 bilateral foi considerado significativo.

**Resultados:**

A pesquisa obteve 142 respostas. Foi relatado que a RMC está disponível para 79% dos respondentes, dos quais 52% raramente ou nunca a utilizam. As indicações mais comuns são cardiomiopatias (84%), pós-operatório de correção de tetralogia de Fallot (81%) e malformações do arco aórtico (53%). A complexidade do exame se correlacionou à relação RMC/cirurgia (Rho = 0,48, IC 95% = 0,32-0,62, p < 0,0001) e ao número de exames de RMC (Rho = 0,52, IC 95% = 0,38-0,64, p < 0,0001). A complexidade da RMC esteve associada à sua realização por cardiologistas pediátricos (RC 2,04, IC 95% 1,2-3,89, p < 0,01). Os principais obstáculos ao uso mais frequente de RMC foram o alto custo (65%), a necessidade de sedação (60%) e o número insuficiente de profissionais qualificados (55%).

**Conclusão:**

A RMC pediátrica não é usada frequentemente no Brasil. A presença de um cardiologista pediátrico a frente dos exames esteve associado ao uso de RMC em pacientes mais complexos. O treinamento de especialistas em RMC pediátrica e a educação dos médicos solicitantes são passos importantes na direção de um uso mais abrangente de RMC no Brasil. (Arq Bras Cardiol. 2021; 116(2):305-312)

## Introdução

A ressonância magnética cardíaca (RMC) é considerada o método padrão-ouro para a avaliação de volumes ventriculares, função sistólica e quantificação de fluxo in vivo.^1-5^ Também pode diagnosticar edema e fibrose com boa concordância com a histologia.^6,7^ Além disso, as imagens de RMC podem ser usadas como matriz para produzir modelos cardíacos tridimensionais, que podem ser usados para ensino, treinamento e planejamento pré-cirúrgico.^8,9^ A combinação dessas qualidades fazem da RMC uma ferramenta poderosa no manejo de pacientes com cardiopatia congênita (CC).

Na última década, a RMC tem sido empregada rotineiramente nos principais centros de cardiologia pediátrica do mundo. Em alguns hospitais, o número de exames de RMC ultrapassa o número de cirurgias cardíacas: No Boston Children’s Hospital, por exemplo, houve 1270 exames de RMC e 947 cirurgias em 2016/2017 (http://www. childrenshospital.org); no Texas Children’s Hospital, houve 941 exames de RMC e 926 cirurgias em 2018 (https://www.texaschildrens.org); e no Hospital for Sick Children em Toronto, aproximadamente 700 exames de RMC e 600 cirurgias são realizados por ano. Esses números se traduzem em uma relação RMC/cirurgia entre 1,02 e 1,34.

No Brasil, números sobre a utilização de RMC não estão disponíveis para consulta pública. A hipótese deste estudo é que a RMC não está amplamente disponível para a população pediátrica e que vem sendo subutilizada. Informações sobre seu uso, suas indicações mais frequentes e os obstáculos à sua utilização, bem como seu papel na tomada de decisões clínico-cirúrgicas, não estão disponíveis. Dados não publicados do Instituto de Cardiologia do Distrito Federal relatam que o número médio anual de exames de RMC pediátricos é de 55, enquanto a média anual de cirurgias cardíacas pediátricas é de 180, resultando em uma relação RMC/cirurgia de 0,31. No Hospital da Criança e Maternidade de São José do Rio Preto, onde aproximadamente 300 cirurgias são realizadas todo ano, apenas 21 exames de RMC foram realizados em 2018 (comunicação pessoal), resultando em uma relação RMC/cirurgia de 0,07. Como esses números não são necessariamente representativos, buscamos obter informações sobre o uso de RMC em crianças em todo o Brasil.

## Métodos

Uma pesquisa foi distribuída a cardiologistas pediátricos e cirurgiões cardíacos em todo o país. Esses indivíduos foram identificados, principalmente, a partir de três grupos de WhatsApp relacionados à área de cardiologia pediátrica: “Grande INCOR” (grupo formado por cardiologistas pediátricos e cirurgiões cardíacos pediátricos do Instituto do Coração de São Paulo [INCOR]), “DCC/CP” (grupo criado pelo Departamento de Cardiopatias Congênitas e Cardiologia Pediátrica da Sociedade Brasileira de Cardiologia, composto por cardiologistas pediátricos e cirurgiões cardíacos pediátricos de todo o país) e “GBCO-Ped” (grupo brasileiro de cardio-oncologia pediátrica que inclui cardiologistas pediátricos com interesse em cardio-oncologia). Esses grupos incluem um total de mais de 350 indivíduos. Contatos pessoais do primeiro autor também foram convidados a participar. Um questionário com 10 perguntas (material suplementar) foi convertido em formato eletrônico via SurveyMonkey (Palo Alto, CA, EUA) e enviado aos participantes. Solicitou-se que os respondentes que trabalhassem em mais de uma instituição, respondessem ao questionário considerando apenas uma delas. A identidade dos respondentes e suas instituições foram preservadas, permitindo, assim a participação de vários respondentes de uma mesma instituição.

**Figura 1:**
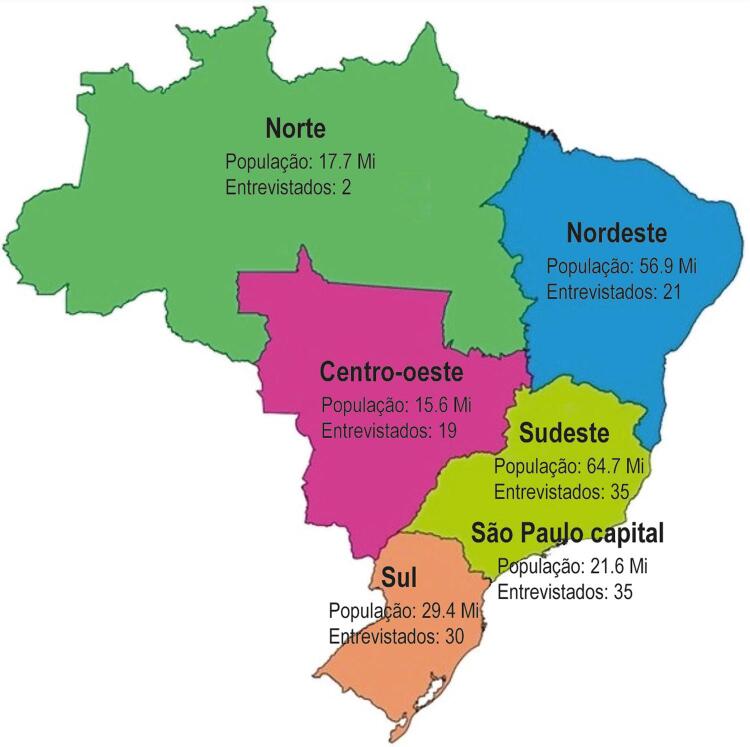
– Distribuição geográfica dos respondentes à pesquisa sobre RMC (adaptado de https://suportegeografico77.blogspot.com/2019/04/mapa-regioes-do-brasil.html).

Devido ao grande número de contatos da cidade de São Paulo (SP), e como a cidade tem metade dos centros de cirurgia cardíaca do estado, com uma população maior do que a das regiões Norte e Centro-Oeste (figura 1), optamos por tratar a cidade de São Paulo como área geográfica separada, ao invés de considerá-la como parte da região Sudeste, para fins deste estudo.^10^

O questionário continha perguntas a respeito dos respondentes (local de trabalho, especialização e número de cirurgias cardíacas realizadas em sua principal instituição de referência), dos serviços de RMC (disponibilidade, especialista a frente dos exames e frequência dos exames), contexto clínico dos pacientes (indicação de RMC, disposição em indicar a operação de Fontan sem cateterismo cardíaco prévio, entre outros) e obstáculos enfrentados para utilizar a RMC nessa população. Questionários contendo apenas respostas sobre os respondentes foram excluídos das análises, já que essas informações sozinhas não teriam acrescentado dados relevantes aos objetivos do estudo. O mesmo questionário foi enviado a um grupo de WhatsApp formado por especialistas em imagem cardíaca pediátrica avançada do Brasil, do qual o autor principal faz parte, com o objetivo de obter dados sobre seus serviços de RMC.

Usamos as respostas à pergunta 3 (número de cirurgias realizadas anualmente) e à pergunta 6 (número de exames de RMC realizados mensalmente) para estimar a relação RMC/cirurgia para cada respondente. Transformamos todas as respostas categóricas individuais em números, já que as opções de resposta eram baseadas em números. Em relação ao número de cirurgias (pergunta 3), se menos de 150 cirurgias são realizadas anualmente, a resposta foi tratada como 100; se entre 150 e 249 cirurgias, a resposta foi tratada como 200; se entre 250 e 349 cirurgias, a resposta foi tratada como 300; e se mais de 350 cirurgias são realizadas por ano, a resposta foi tratada como 500. Em relação ao número de exames de RMC realizados mensalmente (pergunta 6), se nenhum exame de RMC é realizado, a respostas foi tratada como zero; se um ou dois exames de RMC são realizados, a resposta foi tratada como 2; se entre três e cinco exames, a resposta foi tratada como 5; se entre seis e doze exames RMC, a resposta foi tratada como 12; e se treze ou mais exames de RMC são realizados por mês, a resposta foi tratada como 20. Para transformar o índice de exames de RMC mensal em índice anual, os índices foram multiplicados por 12.

Usando esses números, pudemos estimar a relação RMC/cirurgia para cada local, dividindo o número de exames de RMC pelo número de cirurgias cardíacas.

A complexidade da RMC foi estratificada como apresentado abaixo. Procedimentos de alta complexidade envolveram “CC complexa com dúvida diagnóstica”, “pós-operatório de Jatene, Senning ou Mustard”, “anomalia de Ebstein”, “ventrículo direito ou esquerdo hipoplásico” (ventrículos limítrofes), “pré ou pós-operatório de Glenn e Fontan” e “RMC fetal”. Procedimentos de média complexidade envolveram “pós-operatório de tetralogia de Fallot”, “anomalias de situs” e “anomalia do retorno venoso pulmonar”. Procedimentos de baixa complexidade incluíram todas as demais respostas. Essas indicações receberam uma classificação numérica: Alta complexidade = 3, média complexidade = 2 e baixa complexidade = 1. As respostas individuais dos respondentes foram multiplicadas por essas pontuações somadas. A pontuação máxima possível era de 32 e cobria todas as indicações clínicas possíveis. Por exemplo: se alguém usa a RMC apenas para anomalia de Ebstein, ventrículo esquerdo hipoplásico, pós-operatório de tetralogia de Fallot e cardiomiopatia, a classificação de complexidade do serviço de RMC desse respondente é 9.

### Análise Estatística

Considerando que o número de cardiologistas pediátricos registrados no Brasil era de 491 à época (portal.cfm.org.br), o tamanho da amostra estimada para obter um intervalo de confiança de 95% (IC 95%) com uma margem de erro de ± 3%, de acordo com a fórmula de Cochran, era de 95. Variáveis contínuas foram expressas como média ± desvio padrão ou mediana com intervalo interquartil (25-75), conforme apropriado. Para a avaliação da normalidade dos dados, uma ferramenta de cálculo foi utilizada para calcular a área abaixo da curva normal, presumida como aquela onde aproximadamente 95% da área está até 1,96 desvios-padrão da média. Variáveis categóricas foram apresentadas como números e porcentagens. O teste qui-quadrado foi utilizado para avaliar as associações entre variáveis categóricas e razões de chance (RC) ajustadas com seus IC 95%. O teste de posto de Spearman foi utilizado para avaliar as correlações entre variáveis ordinais com distribuições distorcidas. Um p < 0,05 bilateral foi considerado estatisticamente significativo. As análises foram realizadas utilizando o programa StatsDirect, v. 2.7.2 2008 (Cheshire, Reino Unido).

## Resultados

Nossa pesquisa obteve 142 respostas de um total de 364 entrevistados possíveis (índice de resposta 40%). As respostas foram recebidas das seguintes regiões: Norte (1,4%), Nordeste (14,8%), Centro-Oeste (13,4%), SP (24,65%), Sudeste (não incluindo a cidade de São Paulo) (24,65%) e Sul (21,1%) (Figura 1). Devido à pouca participação de respondentes do Norte, essa região não foi representada em análises posteriores, que foram estratificadas por região. A maioria dos respondentes (75%) trabalha nas capitais dos estados. A maioria foi composta de cardiologistas pediátricos (91,5%), seguidos de cirurgiões cardíacos (7%) e cardiologistas de adultos, que também atendem crianças (1,4%). O tamanho dos programas de cirurgia cardíaca onde os médicos de referência trabalham variou de acordo com a região (Tabela 1).

**Tabela 1:** – Resumo dos resultados mais importantes de pesquisa estratificados por região e a cidade de São Paulo

	NE (n = 21)	CO (n = 19)	SE (n = 35)	SP (n = 35)	S (n = 30)
Número de cirurgias cardíacas estimado por centro/ano	200 (200-300)	180 (180-180)	200 (100-307.5)	400 (200-500)	300 (200-480)
RMC disponível - sim	67%	95%	86%	94%	83%
Cardiologista ped. realizando exames de RMC - sim	0	68,5%	28,2%	68,6%	30%
Número de exames de RMC estimado por centro/mês (IQ)	2 (2-2)	4,58 (4,58-4,58)	2 (2-5)	5 (2-12)	2 (2-5)
Classificação da complexidade da RMC: 0-32 (IQ)	7 (5-9)	25 (15-25)	8 (5,5-14)	11 (7-17,8)	9 (6-15)
Razão RMC-cirurgia (DP)	0,11 ± 0,08	0,35 ± 0,27	0,24 ± 0,35	0,36 ± 0,35	0,14 ± 0,1

RMC: ressonância magnética cardíaca; Regiões NE: Nordeste; CO: Centro-oeste; SE: Sudeste; SP: São Paulo; S: Sul.

Um total de 79% dos respondentes relatou que a RMC está ‘disponível’. Esse índice variou amplamente de acordo com a região (Tabela 1), sendo que respondentes de Goiânia, Belém e Palmas relataram que a RMC não está disponível para eles. A RMC está disponível para 68% dos médicos que trabalham fora das capitais dos estados.

**Figura 2:**
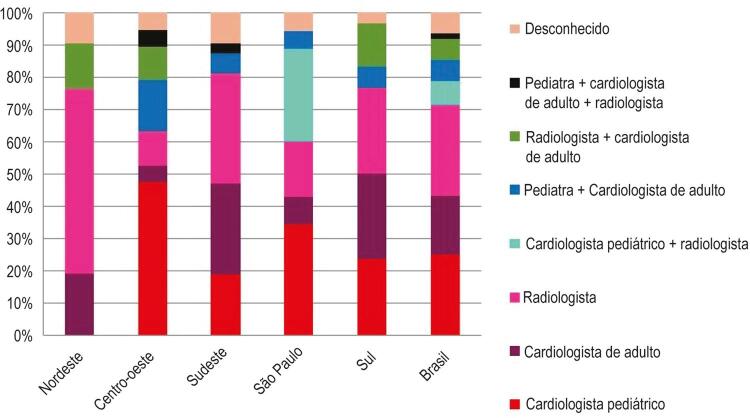
– Tipos de especialistas que realizam RMC pediátrica no Brasil de acordo com a região. Ped: pediatra

No Brasil, a RMC é realizada por radiologistas, seguida de cardiologistas pediátricos. Em algumas áreas, cardiologistas pediátricos a frente dos exames de RMC são raros ou inexistentes (Figura 2). A maioria dos respondentes (61%) relatou que raramente ou nunca usam a RMC, enquanto 15% relataram que a usam frequentemente. A frequência do uso de RMC variou por região geográfica (Tabela 1). A relação RMC/cirurgia no Brasil foi estimada entre 0,22 ± 0,27, variando de acordo com a região (Tabela 1).

### Indicações de Ressonância Magnética Cardíaca

Nossa pesquisa mostrou que as três indicações mais comuns para RMC são cardiomiopatias (84%), pós-operatório de tetralogia de Fallot (81%) e malformações do arco aórtico (53%) (Tabela 2). Também foram encontradas diferenças regionais na complexidade da RMC (Tabela 1). Encontramos correlações entre a complexidade do exame e a relação RMC/cirurgia (Rho = 0,48, IC 95% = 0,32-0,62, p < 0,0001) e entre complexidade do exame e número de exames de RMC (Rho = 0,52, IC 95% = 0,38-0,64, p < 0,0001). Nenhuma correlação foi encontrada entre a complexidade da RMC e o número de cirurgias (p = 0,73).

**Tabela 2:** – Indicações de RMC para crianças no Brasil

Indicação de RMC	Respostas afirmativas (%)
Cardiomiopatias	84
Status pós-reparo de tetralogia de Fallot	81
Doença do arco aórtico	53
DCC complexa	46
Anomalia de Ebstein	45
Tumores cardíacos	44
Hipoplasia ventricular direita ou esquerda	36
Pré- e/ou pós-Glenn e Fontan	36
Status de pós-reparo de transposição das grandes artérias	34
Anomalias coronárias	32
Retorno venoso pulmonar	31
Síndrome de Marfan	18
Status pós-transplante cardíaco	16
Cardiotoxicidade	14
Anomalias de situs	14
Arterite de Takayasu	14
RMC fetal	3

RMC: ressonância magnética cardíaca; DCC: doença cardíaca congênita.

Também encontramos uma correlação positiva entre alta complexidade da RMC (≥ 18) e a presença de um cardiologista pediátrico a frente dos exames (RC 2,14, IC 95% 1,2-3,89, p 0,01), bem como uma correlação inversa entre alta complexidade e realização dos exames por um cardiologista de adultos (RC 0,42, IC 95% 0,19-0,89, p 0,03).

### Necessidade de Cateterismo de Rotina Antes da Operação de Fontan

Um número relativamente alto de respondentes (43%) consideraria indicar operação de Fontan sem cateterismo cardíaco (CATE) prévio em pacientes selecionados, com base em achados do ecocardiograma (ECO) e da RMC.

Essa disposição em indicar a operação de Fontan sem CATE prévio está inversamente correlacionada ao tamanho do programa de cirurgia cardíaca (RC 0,6, IC 95% 0,38-0,93, p 0,02) e à falta de confiança na avaliação de fluxos pela RMC (RC 0,42, IC 95% 0,31-0,56, p < 0,0001).

### Confiança no Ecocardiograma e Cateterismo Cardíaco Versus RMC para Avaliação de Fluxos

Entre os respondentes, 74% relataram confiar mais nas avaliações de fluxo por ECO e/ou CATE do que em avaliações de fluxo pela RMC.

Não identificamos associações entre confiança na avaliação de fluxos pela RMC, número de exames de RMC realizados mensalmente, uso de RMC para pré ou pós-operatório de Glenn e Fontan, ou tipo de especialistas à frente dos exames.

### Obstáculos ao Uso de RMC

De acordo com os respondentes, os principais obstáculos ao uso mais frequente de RMC foram o alto custo (65%), a necessidade de sedação (60%) e um número insuficiente de profissionais qualificados (55%; Tabela 3). Um dos respondentes mencionou que, em sua cidade, a RMC está disponível apenas para adultos.

**Tabela 3:** – Obstáculos ao uso de RMC

Obstáculos	%
Custo	65
Necessidade de sedação	60
Número insuficiente de profissionais qualificados	55
Tempo de espera	40
Conhecimento limitado e pouca promoção da RMC	24
Inferioridade à angiografia por tomografia computadorizada	21
Credibilidade limitada	16
Outros	5

RMC: ressonância magnética cardíaca.

## Discussão

Até onde sabemos, esta é a primeira pesquisa a avaliar o uso de RMC em crianças no Brasil. Foram encontradas diferenças regionais em vários aspectos da prática com RMC: especialistas que realizam a RMC, indicações para RMC, relação RMC/cirurgia, etc. Isso corrobora nossa impressão de que o uso de RMC é heterogêneo em todo o país. Este estudo identificou que, em comparação com os principais centros na Europa e América do Norte, a RMC não é suficientemente utilizada no Brasil, em relação ao número de cirurgias. Este estudo sugere que a razão para esse achado pode ser multifatorial.

O custo foi citado como o principal obstáculo ao uso mais frequente de RMC. Entretanto, considerando a disponibilidade relativamente alta de RMC pediátrica relatada nesta pesquisa, a resposta pode, por sua vez, refletir uma dificuldade de acesso a RMC. No Brasil, a maioria dos pacientes com cardiopatias congênitas são tratados em instituições públicas, que geralmente atuam com verbas muito restritas. Em evidente contraste com as necessidades de crianças e adolescentes com cardiopatias congênitas, de acordo com os dados de 2012 do Ministério da Saúde do Brasil, havia 84 aparelhos de ressonância magnética disponíveis para uso do Sistema Único de Saúde, e 1.263 aparelhos de ressonância magnética disponíveis para o sistema particular de saúde. A carência de aparelhos disponíveis é acentuada, ainda mais, pela necessidade de compartilhar o aparelho com outras especialidades. Na prática do autor principal, por exemplo, há apenas um aparelho disponível para exames de RMC pediátricos ou congênitos uma manhã por semana. Portanto, a relação RMC/cirurgia mais alta que pode ser atingida é de 0,6:1. Para fins de comparação, no Hospital for Sick Children em Toronto, há seis aparelhos para uso clínico, um deles dedicado exclusivamente a exames de RMC pediátrica por 6 horas diariamente. Outra explicação possível para os obstáculos da ‘disponibilidade de aparelhos’ é a questão do reembolso. Em nossa instituição, o sistema público de saúde reembolsa exames de RMC com R$403, enquanto uma tomografia computadorizada (TC) é reembolsada com R$322, e um ECO é reembolsado com R$165. Em termos relativos, essas diferenças não são diferentes às existentes nos EUA. Portanto, essa não parece ser uma razão adequada para a subutilização da RMC em relação ao ECO e à TC. Em números absolutos, entretanto, o reembolso para todos os tipos de estudos de imagem é insuficiente. (http://www2.ebserh.gov. br/documents).

O segundo obstáculo mais importante a uma aplicação mais frequente da RMC foi identificado como sendo a necessidade de sedação ou anestesia, geralmente solicitada para crianças abaixo dos 8 anos de idade. Essa é uma preocupação frequente para os médicos dos pacientes e os pais das crianças. Entretanto, é importante enfatizar que o número de eventos adversos sofridos por crianças como resultado da sedação para RMC é muito baixo.^11^ Outro ponto a ser considerado é que a RMC é uma técnica não invasiva e que não expõe pacientes à radiação ionizante, caso do CATE cardíaco ou da TC.^12^ De qualquer forma, é importante mencionar que são tomadas precauções para evitar a sedação e que há muitas estratégias que podem ser adotadas para esse fim.^13-16^

O terceiro obstáculo mais importante ao uso mais frequente de RMC foi o número insuficiente de profissionais qualificados. A Sociedade de Ressonância Magnética Cardiovascular estratifica o treinamento para RMC em três níveis: 1. treinamento básico, 2. treinamento especializado e 3. treinamento avançado.^17^ Existe uma obrigatoriedade de se realizar pelo menos 150 exames de RMC por ano para obtenção do certificado de nível 2, que constitui o nível mínimo exigido na Europa para que cardiologistas pediátricos realizem RMC.^17-19^ No Brasil, não há regulamentação a esse respeito, mas não seria irracional buscar o padrão de referência europeu. Pode ser simplesmente uma questão de tempo até que a especialização em RMC pediátrica/congênita evolua em nosso país e que profissionais qualificados estejam disponíveis no mercado. É interessante observar que a força tarefa COCATS 4, reconhece que as habilidades em identificar cardiopatias congênitas simples e complexas em adultos, incluindo a quantificação de shunts cardíacos, geralmente são adquiridas após 36 meses de exposição aos casos de RMC em um serviço de RMC geral.^20^ Portanto, recomenda-se “concentrar” a experiência de aprendizagem fazendo o treinamento em instituições pediátricas especializadas. Na Europa e na América do Norte, por exemplo, são oferecidas bolsas para treinamento em RMC pediátrica/congênita em locais onde são realizados um alto número de exames pediátricos e congênitos. Os programas geralmente são localizados dentro de hospitais pediátricos, com um grande volume de cirurgias cardíacas. Muito frequentemente, esses locais têm cardiologistas pediátricos como membros integrantes de suas equipes de exames de imagem. No Brasil, esse cenário existe apenas na cidade de São Paulo, onde o número de cirurgias é alto e onde há cardiologistas pediátricos rotineiramente envolvidos na prática de RMC pediátrica. No momento atual, portanto, nos parece que o treinamento de radiologistas e cardiologistas para se tornarem especialistas em exames de RMC pediátricos, deveria envolver algum treinamento em centros acadêmicos na Europa e na América do Norte.

Quase todos os respondentes já solicitaram exames de RMC para pacientes com cardiomiopatia ou pós-operatório de tetralogia de Fallot, indicações de baixa e média complexidade. Exames mais complexos são realizados em centros com as relações RMC/cirurgia mais altas, especialmente quando cardiologistas pediátricos estão envolvidos nos exames. Por outro lado, não se identificou necessariamente a relação entre a complexidade dos exames e o número de cirurgias realizadas. É provável que locais que ofereçam RMC guiadas por cardiologistas pediátricos atraiam os casos mais complexos, talvez por estarem mais preparados para aceitar esses casos, ou por esses serviços estarem mais integrados à prática cardiológica.

Na ampla maioria dos centros mundiais, é rotina que pacientes com coração univentricular passem por um CATE cardíaco pré-Fontan para avaliar a anatomia arterial pulmonar, medir a pressão do leito arterial pulmonar e estimar a resistência vascular pulmonar. Alguns especialistas têm encorajado o uso apenas de RMC nesses casos, sendo o CATE destinado apenas a pacientes de alto risco.^21-25^ Nessa pesquisa, 43% dos respondentes relataram que estariam dispostos a indicar uma operação de Fontan apenas com informações de ECO e RMC, evitando, assim, o CATE em pacientes considerados de risco padrão, embora tenhamos observado uma grande variação nas práticas entre regiões.

### Limitações do Estudo

Reconhecemos que, embora o tamanho da amostra estimada seja suficiente para responder nossas perguntas, o alto número de não respondentes, algo esperado nesse tipo de pesquisa, é uma fonte de viés em potencial. A limitação mais importante da pesquisa, entretanto, foi o fato de que o número real de exames de RMC pediátricas por centro continua desconhecido. Tentamos obter esses números de radiologistas e cardiologistas que são responsáveis ou fazem parte de equipes de imagem dos maiores programas de cirurgia cardíaca pediátrica no Brasil, mas apenas uma instituição concordou em compartilhar seus dados. Portanto, as relações RMC/cirurgia encontradas, e todas as associações entre o número de exames de RMC e número de cirurgias foram simples estimativas, e podem não ser, até certo ponto, representativas da situação no Brasil. Para mitigar incertezas e melhorar a comparabilidade entre instituições, oferecemos opções de resposta que incluíram faixas de números ao invés de números precisos. É possível que tenhamos recebido respostas de vários indivíduos da mesma instituição e que essas respostas possam, até certo ponto, ter expressado estimativas individuais e não dados institucionais concretos, e, também, que algumas instituições possam ter sido desproporcionalmente mais representadas no grupo de respondentes. Da mesma forma, não conseguimos avaliar a disponibilidade da RMC e outros aspectos na região Norte do Brasil, devido à baixa participação de colegas dessa área.

## Conclusões

A RMC pediátrica está disponível para aproximadamente 2/3 dos médicos, mas não é muito frequentemente utilizada no Brasil. Os exames geralmente são realizados por radiologistas. A RMC é mais comumente indicada para cardiomiopatias e pós-operatório de tetralogia de Fallot. A presença de um cardiologista pediátrico a frente dos exames de RMC está associada ao uso de RMC em pacientes mais complexos. Os obstáculos ao uso mais frequente da RMC foram custo, necessidade de sedação e falta de profissionais qualificados. Um maior treinamento de especialistas em RMC pediátrica e uma maior educação sobre o método aos médicos solicitadores dos exames são passos importantes para o aumento do uso de RMC no Brasil. A colaboração entre instituições é recomendada e necessária a fim de se ter um cenário mais fidedigno sobre o uso da RMC na população pediátrica no Brasil.
