# Supplementary material for: Uso Atual de Ressonância Magnética Cardíaca Pediátrica no Brasil
Source: Arq Bras Cardiol. 2021 Feb 19;116(2):305–12. [Article in Portuguese] doi: 10.36660/abc.20190860 (PMC7909959; doi:10.36660/abc.20190860)
Supplement: Translation

# Translation: Current Use of Pediatric Cardiac Magnetic Resonance Imaging in Brazil

**Authors:** Marcelo Felipe Kozak, Jorge Yussef Afiune, Lars Grosse-Wortmann

**Affiliations:** 1 BrazilInstituto de Cardiologia do Distrito Federal - Cardiologia Pediátrica, Brasília, DF - Brazil; 2 BrazilHospital da Criança de Brasília José de Alencar – Ecocardiografia, Brasília, DF - Brazil; 3 USADoernbecher Children’s Hospital, Oregon Health and Science University - Division of Pediatric Cardiology, Department of Pediatrics, Portland, Oregon - USA; 4 CanadaThe Hospital for Sick Children, University of Toronto - Department of Pediatrics, Toronto, Ontario – Canada

**Keywords:** Pediatrics, Heart Defects,Congenital/surgery, Diagnostic Techniques Procedures, Magnetic Resonance Imaging, Cardiac Catheterization

## Abstract

**Background:**

Data on the use of cardiac magnetic resonance imaging (CMR) on children in Brazil is lacking.

**Objectives:**

This study sought to provide information on current pediatric CMR practices in Brazil.

**Methods:**

A questionnaire was sent out to referring physicians around the country. It covered information on the respondents, their CMR practices, the clinical context of the patients, and barriers to CMR use among children. For statistical analysis, two-sided p < 0.05 was considered significant.

**Results:**

The survey received 142 replies. CMR was reported to be available to 79% of the respondents, of whom, 52% rarely or never use CMR. The most common indications were found to be cardiomyopathies (84%), status of post-tetralogy of Fallot repair (81%), and aortic arch malformations (53%). Exam complexity correlated with CMR-to-surgery ratio (Rho = 0.48, 95% CI = 0.32-0.62, p < 0.0001) and with the number of CMR exams (Rho = 0.52, 95% CI = 0.38-0.64, p < 0.0001). Further, a high CMR complexity score was associated with pediatric cardiologists conducting the exams (OR 2.14, 95% CI 1.2-3.89, p < 0.01). The main barriers to a more frequent use of CMR were its high cost (65%), the need for sedation (60%), and an insufficient number of qualified professionals (55%).

**Conclusion:**

Pediatric CMR is not used frequently in Brazil. The presence of a pediatric cardiologist who can perform CMR exams is associated with CMR use on more complex patients. Training pediatric CMR specialists and educating referring providers are important steps toward a broader use of CMR in Brazil. (Arq Bras Cardiol. 2021; 116(2):305-312)

## Introduction

Cardiac magnetic resonance imaging (CMR) is considered the gold standard for the assessment of ventricular volumes, systolic function, and *in vivo* flow quantification.^1-5^ It can also diagnose edema and fibrosis with good agreement with histology.^6,7^ In addition, CMR images may be used as a matrix source in order to produce three-dimensional cardiac models that can be used for teaching, training, and preoperative planning.^8,9^ The combination of these advantages makes CMR a powerful tool in the management of patients with congenital heart disease (CHD).

Over the last decade, CMR has been routinely employed at leading pediatric cardiology centers worldwide. In some hospitals, the number of CMR exams exceeds the number of cardiac surgeries. At Boston Children’s Hospital, for example, there were 1,270 CMR exams and 947 surgeries in 2016/2017 (http://www.childrenshospital.org); at Texas Children’s Hospital, there were 941 CMR exams and 926 surgeries in 2018 (https://www.texaschildrens.org); and at the Hospital for Sick Children in Toronto, roughly 700 CMR exams and 600 surgeries are performed each year. These figures result in a CMR-to-surgery ratio between 1.02 and 1.34.

In Brazil, numbers on the use of CMR are not publicly available. It was hypothesized that CMR is not widely available for the pediatric population and that it is being underused. Information on CMR use, the most frequent indications for and barriers to its use, as well as the role of CMR in cardiac and surgical decision-making are not available. Unpublished data from the Heart Institute of Distrito Federal show that their average annual number of pediatric CMR exams is 55, while their average annual number of pediatric cardiac surgeries is 180, resulting in a CMR-to-surgery ratio of 0.31. At the Children’s and Maternity Hospital of São Jose do Rio Preto, where approximately 300 surgeries are performed each year, only 21 CMR exams were performed in 2018 (personal communication), resulting in a CMR-to-surgery ratio of 0.07. Because these numbers are not necessarily representative, the present study sought to obtain information on the use of CMR on children in Brazil.

## Methods

A survey was distributed to pediatric cardiologists and cardiac surgeons from across Brazil. These individuals were identified mainly from three WhatsApp groups related to the field of pediatric cardiology: “Grande INCOR” (a group consisting of current and prior pediatric cardiologists and pediatric cardiac surgeons from the Heart Institute of São Paulo [INCOR]), “DCC/CP” (a group created by the Department of Congenital Heart Disease and Pediatric Cardiology of the Brazilian Society of Cardiology, including pediatric cardiologists and pediatric cardiac surgeons from around the country), and “GBCO-Ped” (a Brazilian pediatric cardio-oncology group, which includes pediatric cardiologists with any interest in cardio-oncology). These groups include a total of more than 350 individuals. The first author’s individual contacts were also asked to participate. A questionnaire with 10 questions (Supplementary Material) was converted into electronic format via SurveyMonkey (Palo Alto, CA, USA) and sent out to the aforementioned groups. Respondents who worked in more than one institution were asked to provide answers regarding only one of them. The identity of the respondents and their institutions remained anonymous, thus allowing for multiple respondents from a single institution.

Due to the large number of contacts from the city of São Paulo (SP) and because it is a city with half of the cardiac surgery centers in the state of São Paulo with a population that is larger than the regions North and Central-West (Figure 1), SP, for the purpose of this study, was thus treated as a separate geographic area, rather than part of the Southeast region.^10^

**Figure 1:** – CMR survey respondents’ geographic distribution (adapted from https://suportegeografico77.blogspot.com/2019/04/mapa-regioes-do-brasil.html).

The questionnaire contained questions covering the respondents (workplace, expertise, and number of cardiac surgeries performed at his/her main reference institution), CMR practices (availability, specialist performing the exams, and frequency of CMR exams), clinical contexts of the patients (CMR indication, readiness to proceed with Fontan operation without a prior cardiac catheterization, among others), and barriers faced when using CMR in this population. Questionnaires containing only answers about the respondents were excluded from the analysis, since this information alone would have not added relevant data to the objectives of the study. The same questionnaire was sent out to a WhatsApp group of cardiac imagers with pediatric/congenital expertise in Brazil, in which the first author participates, with the objective of obtaining data on their numbers of CMR exams and their CMR practices.

This study used the answers to Questions 3 (the number of surgeries performed annually) and 6 (the number of CMR exams performed monthly) to estimate the CMR-to-surgery ratio for each respondent. Each individual categorical answer was transformed into a number, since the answer options were number based. With regard to the number of surgeries (Question 3), if fewer than 150 surgeries were performed annually, the answer was treated as 100; if between 150 and 249 surgeries were performed, the answer was treated as 200; if between 250 and 349 surgeries were performed, the answer was treated as 300; and if 350 or more surgeries were performed a year, the answer was treated as 500. With regard to the number of CMR exams performed monthly (Question 6), if no CMR exams were performed, the answer was treated as zero; if one or two CMR exams were performed, the answer was treated as 2; if three to five CMR exams were performed, the answer was treated as 5; if six to twelve CMR exams were performed, the answer was treated as 12; and if thirteen or more CMR exams were performed a month, the answer was treated as 20. These rates were multiplied by twelve in order to transform the monthly rate of CMR exams into an annual rate.

Using these numbers, it was possible to estimate the CMR-to-surgery ratio for each site by dividing the number of CMR exams by the number of cardiac surgeries.

The complexity of the CMR was stratified as follows. High complexity procedures involved “complex CHD with a diagnostic query,” “status following Jatene, Senning, or Mustard operations”, “Ebstein’s anomaly,” “hypoplastic right or left ventricles” (borderline ventricles), “status pre- or post- Glenn and Fontan operations,” and “fetal CMR”. Medium complexity procedures involved “status post-tetralogy of Fallot repair,” “situs anomalies,” and “pulmonary venous return anomalies”. Low complexity procedures defined all of the remaining answers. These indications received a numerical score: High complexity = 3, medium complexity = 2, and low complexity = 1. The respondents’ individual answers were multiplied by these scores added together. The maximum possible score was 32, which covered all possible clinical indications. For instance: if someone has used CMR only for Ebstein´s anomaly, hypoplastic left ventricle, status post-tetralogy of Fallor repair and cardiomyopathy, this respondent´s score is 9.

### Statistical Analyses

Considering that the number of registered pediatric cardiologists in Brazil is 491 (portal.cfm.org.br), the sample size estimated to have a 95% confidence interval (CI) and a margin of error of ± 3%, which, according to Cochran´s formula, was 95. Continuous variables were expressed as means plus or minus standard deviation or median with interquartile range (25-75), as appropriate. For the assessment of data normality, a calculation tool was employed to calculate the area under a normal curve, assumed to be when approximately 95% of the area was within 1.96 standard deviations of the mean. Categorical variables were summarized as numbers and percentages. The chi-square test was used to assess associations between categorical variables and adjusted odds ratios (ORs) with their 95% CIs. Spearman’s rank test was used to assess correlations between ordinal variables with skewed distributions. Two-sided p < 0.05 was considered statistically significant. Analyses were performed using StatsDirect, v. 2.7.2 2008 (Cheshire, UK).

## Results

Our survey produced 142 responses for a response rate of 142/364 (40%). In summary, responses were received from the following regions: the North (1.4%), the Northeast (14.8%), the Central-West (13.4%), SP (24.65%), the Southeast (not including São Paulo) (24.65%), and the South (21.1%) (Figure 1). Due to the limited participation of respondents from the North, this region was not represented in further analyses that were stratified by region. Most of the respondents (75%) worked in state capitals and were most commonly pediatric cardiologists (91.5%), followed by cardiac surgeons (7%), and adult cardiologists who also treat children (1.4%). The size of the cardiac surgery programs where the referring physicians worked varied by region (Table 1).

**Table 1:** – Summary of the most important survey results stratified by region and the city of São Paulo

	NE (n = 21)	CW (n = 19)	SE (n = 35)	SP (n = 35)	S (n = 30)
Estimated number of cardiac surgeries per center/year	200 (200-300)	180 (180-180)	200 (100-307.5)	400 (200-500)	300 (200-480)
CMR available – yes	67%	95%	86%	94%	83%
Ped cardiologist performing CMR exams – yes	0	68.5%	28.2%	68.6%	30%
Estimated number of CMR exams per center/month (IQ)	2 (2-2)	4.58 (4.58-4.58)	2 (2-5)	5 (2-12)	2 (2-5)
CMR complexity score:0-32 (IQ)	7 (5-9)	25 (15-25)	8 (5.5-14)	11 (7-17.8)	9 (6-15)
CMR-to-surgery ratio (SD)	0.11 ± 0.08	0.35 ± 0.27	0.24 ± 0.35	0.36 ± 0.35	0.14 ± 0.1

CMR: cardiac magnetic resonance imaging; Regions: NE: Northeast; CW: Central-West; SE: Southeast; SP: São Paulo; S: South.

CMR was reported as ‘available’ by 79% of the respondents. This rate varied widely by region (Table 1), with respondents from Goiânia, Belém, and Palmas reporting that CMR was not available to them. CMR was available to 68% of the physicians who work outside of capital cities.

In Brazil, CMR is performed by radiologists, followed by pediatric cardiologists. In some areas, pediatric cardiologists who perform CMR exams are rare or non-existent (Figure 2).

**Figure 2:** – Types of specialists performing pediatric CMR in Brazil by region.

Most of the respondents (61%) reported that they rarely or never use CMR, while 15% reported that they use it often. The frequency of CMR use varied by geographic region (Table 1). The overall Brazilian CMR-to-surgery ratio was estimated to be 0.22 ± 0.27, which also varied by region (Table 1).

### Cardiac Magnetic Resonance Indications

Our survey showed that the three most common indications for CMR were cardiomyopathies (84%), status post-tetralogy of Fallot repair (81%), and aortic arch malformations (53%) (Table 2). Regional differences on CMR complexity were also found (Table 1).

**Table 2:** – CMR indications for children in Brazil

CMR Indication	Affirmative Answers (%)
Cardiomyopathies	84
Status post-tetralogy of Fallot repair	81
Aortic arch disease	53
Complex CHD	46
Ebstein’s anomaly	45
Cardiac tumors	44
Right or left ventricular hypoplasia	36
Pre- and or post-Glenn and Fontan	36
Status post-transposition of the great arteries repair	34
Coronary anomalies	32
Pulmonary venous return	31
Marfan	18
Status post-heart transplant	16
Cardiotoxicity	14
Situs anomalies	14
Takayasu arteritis	14
Fetal CMR	3

CMR: cardiac magnetic resonance imaging; CHD: congenital heart disease.

Our findings showed correlations between exam complexity and CMR-to-surgery ratio (Rho = 0.48, 95% CI = 0.32-0.62, p < 0.0001), as well as between exam complexity and the number of CMR exams (Rho = 0.52, 95% CI = 0.38-0.64, p < 0.0001). No correlation was found between CMR complexity and number of surgeries (p = 0.73).

The results also pointed to a positive correlation between a high CMR complexity score (≥ 18) and whether the exam was performed by a pediatric cardiologist (OR 2.14, 95% CI 1.2-3.89, p 0.01), as well as an inverse correlation between high complexity and performance by an adult cardiologist (OR 0.42, 95% CI 0.19-0.89, p 0.03).

### Need for Routine Catheterization prior to Fontan

A relatively high number of the respondents (43%) would consider ordering a Fontan operation without prior cardiac catheterization (cath) in selected patients, based on the combination of echocardiography (echo) and CMR assessments.

It was observed that the willingness to proceed with the Fontan completion without prior cath correlated inversely with the size of the cardiac surgery program (OR 0.6, 95% CI 0.38-0.93, p 0.02) and with a lack of confidence in CMR flow assessment (OR 0.42, 95% CI 0.31-0.56, p < 0.0001).

### Confidence in Echocardiography and Cardiac Catheterization versus CMR for Flow Assessments

Of the respondents, 74% reported relying more on flow assessments by echo and/or cath than on the flow assessment by CMR.

No associations were found between confidence in echo/cath or CMR flow assessment and the number of CMR exams performed monthly, the use of CMR for pre- or post- Glenn and Fontan surgeries, or the type of specialist performing the CMR exams.

### Barriers to CMR Use

According to the respondents, the main barriers to the more frequent use of CMR are its high cost (65%), the need for sedation or anesthesia (60%), and the insufficient number of qualified professionals (55%; Table 3). One of the respondents mentioned that CMR is available only for adults in the city where he/she is employed.

**Table 3:** – Barriers to CMR use

Barriers	%
Cost	65
Need for sedation	60
Insufficient number of qualified professionals	55
Waiting time	40
Limited education on and promotion of CMR	24
Inferiority to computed tomography angiography	21
Limited credibility	16
Other	5

CMR: cardiac magnetic resonance imaging.

## Discussion

To the best of our knowledge, this is the first survey to assess the use of CMR in children in Brazil. Regional differences were identified in many aspects of CMR practice: specialist performing CMR, CMR indications, CMR-to-surgery ratio, among others. This corroborates our impression that CMR use is diverse throughout the country. This study found that, in comparison to the leading centers in Europe and North America, CMR is very underused in Brazil as regards the number of surgeries. This study suggests that the reason for this finding may be multifactorial.

Cost was cited as the main barrier to more frequent CMR use. However, considering the relatively high availability of pediatric CMR reported in this survey, this answer might instead be reflecting a difficulty of access to CMR. In Brazil, most patients with CHD are treated at public facilities, which usually operate with very restricted budgets. In stark contrast to the needs of children and adolescents with CHD, according to 2012 data from the Brazilian Ministry of Health, 84 MRI scanners were available for the country’s publicly funded health insurance, and 1,263 MRI scanners were available for private practice. The paucity of available scan time is further worsened by the need to share the magnet with other pediatric and/or adult specialties. At the first author’s practice, for example, there is only one scanner, which is available for routine pediatric or congenital CMR exams only one morning per week. Therefore, the highest CMR-to-surgery ratio we can achieve is 0.6:1. For the sake of comparison, at the Hospital for Sick Children in Toronto, there are six scanners for clinical use, one of which is dedicated exclusively to pediatric CMR exams for 6 hours every day. Another possible explanation for the reported barrier of ‘scanner availability’ is the question of reimbursement. At our site, the public health care system reimburses CMR exams with BRL$403 (US$103), while BRL$322 (US$83) is reimbursed for a computed tomography (CT), and BRL$165 (US$42) is reimbursed for an echo. In relative terms, these differences are not dissimilar to those in the US. Therefore, it does not seem to be a suitable reason for the underuse of CMR as regards echo and CT. In absolute figures, however, the reimbursement for all types of imaging studies is insufficient (http://www2.ebserh.gov.br/documents).

The second most important barrier to a more frequent application of CMR was found to be the need for sedation or anesthesia, which is usually requested for children under 8 years of age. This is often a concern for both the referring physician and his or her parents. However, it is important to emphasize that the number of adverse events experienced by children as a result of sedation for CMR is very low.^11^ Another point to consider is that CMR is a non-invasive technique that does not expose patients to ionizing radiation, as is the case with the use of cardiac cath or CT.^12^ However, it is important to mention that measures are taken to avoid sedation and that there are many strategies that can be used to this end.^13-16^

The third most important barrier to a more frequent use of CMR was reported to be an insufficient number of qualified professionals. The Society for Cardiovascular Magnetic Resonance stratifies CMR training into three levels: 1. basic training, 2. specialized training, and 3. advanced training.^17^ There is a requirement to perform at least 150 CMR cases a year to obtain the level 2 certificate which constitutes the minimum level required in Europe for pediatric cardiologists to perform a CMR.^17-19^ In Brazil, there is no regulation on this matter, but it would not seem unreasonable to seek the implementation of European standards. It may be simply a matter of time before specialization in pediatric/congenital CMR evolves and more qualified professionals are available in the market. Interestingly, the COCATS 4 task force recognizes that skills to identify basic and complex CHD in adults, including quantification of shunts, are usually acquired after 36 months of exposure to CMR cases in a general CMR setting.^20^ Therefore, it is advisable to “concentrate” the learning experience by training in specialized pediatric facilities. In Europe and North America, for example, pediatric/congenital CMR fellowships are offered at sites that perform a high number of pediatric and congenital exams. These programs are usually located within pediatric hospitals with a high volume of cardiac surgeries. More often than not, these sites have a pediatric cardiologist as an integral member of their imaging teams. In Brazil, this setup exists only in the city of São Paulo, where the number of surgeries is high and where there are pediatric cardiologists routinely involved in the pediatric CMR practice. At the present time, training radiologists and cardiologists to become experts in pediatric CMR exams realistically involves physicians training at academic centers in Europe and North America.

Almost all of the respondents had previously requested CMR exams for patients with cardiomyopathy or after tetralogy of Fallot repair, indications of low and medium complexity. More complex CMR exams are performed in centers with the highest CMR-to-surgery ratios and especially when pediatric cardiologists are involved in scanning. On the other hand, the complexity of the exams was not necessarily found to be related to the number of surgeries performed. It seems possible that sites that offer CMR and that employ pediatric cardiologists attract more complicated cases, perhaps because they are more prepared to take on these cases or because these services tend to be more integrated into cardiology practice.

In the vast majority of centers worldwide, it is routine for a patient with a univentricular heart to receive a pre-Fontan cardiac cath for the assessment of the pulmonary arterial anatomy, measurement of the pressure of the pulmonary arterial bed, and estimation of pulmonary vascular resistance. Some experts have encouraged the routine sole use of CMR in pre-Fontan patients, with cath reserved for high-risk patients.^21-25^ In this survey, 43% of respondents reported that they would be willing to order a Fontan operation only with information from echo and CMR, thus avoiding cath in patients who are deemed to be standard risk, although there is a large variation in practices between regions.

### Study Limitations

The authors of this study recognize that, although the sample size estimated was sufficient to address our questions, the high number of non-respondents, something expected in these kinds of surveys, was a potential source of bias. The most important limitation of our survey, however, was the fact that the actual number of pediatric CMR exams per center remains unknown. We tried to obtain these numbers from radiologists and cardiologists who lead or are part of the imaging teams of the largest pediatric cardiac surgery programs in Brazil, but only one site agreed to share its CMR statistics. Therefore, the CMR-to-surgery ratios found and all associations between the number of CMR exams and the number of surgeries were mere estimates, and may not be, to some extent, representative of Brazil. To mitigate uncertainties and improve comparability between sites, our study offered answer options that included ranges rather than precise numbers. It is possible that we have received responses from several individuals from the same institution and that these responses may, to a certain extent, have expressed individual estimates rather than concrete institutional data and that some institutions may have been overrepresented in the pool of respondents. It was also impossible to evaluate the availability of CMR and other aspects of CMR in the North of Brazil due to the low participation of colleagues from this area.

## Conclusions

Pediatric CMR is available to approximately 2/3 of practitioners, but it is rarely used in Brazil. Exams are most often performed by radiologists. CMR is most commonly obtained for cardiomyopathies and after tetralogy of Fallot repair. The presence of a pediatric cardiologist to perform the CMR exams is associated with CMR use in more complex patients. Obstacles to a more frequent use of CMR are cost, the need for sedation, and a lack of qualified professionals. Training pediatric CMR specialists and educating referring providers are important steps toward an increased use of CMR in Brazil. Collaboration between institutions is advisable and necessary in order to have a better picture of the use of CMR in the pediatric population in Brazil.
